# Supplementary material for: Macroinvertebrate Response to Internal Nutrient Loading Increases in Shallow Eutrophic Lakes
Source: Biology (Basel). 2023 Sep 18;12(9):1247. doi: 10.3390/biology12091247 (PMC10525641; doi:10.3390/biology12091247)
Supplement: Supplementary file 1 [file biology-12-01247-s001.zip › biology-2520325-supplementary.pdf]

Macroinvertebrate response to internal nutrient loading increases in shallow eutrophic lakes

Kai Peng[1], Rui Dong[1,2], Boqiang Qin[1,3], Yongjiu Cai[1,4*], Jianming Deng[1], Zhijun Gong[1,4]

1 Taihu Laboratory for Lake Ecosystem Research, State Key Laboratory of Lake Science and Environment, Nanjing Institute of Geography and Limnology, Chinese Academy of Sciences, 73 East Beijing Road, Nanjing 210008, P.R. China; kpeng@niglas.ac.cn (K.P); dongrui21@mails.ucas.ac.cn (R.D); qinbq@niglas.ac.cn (B.Q); caiyj@niglas.ac.cn (Y.C); jmdeng@niglas.ac.cn (J.D); zjgong@niglas.ac.cn (Z.G)

2University of Chinese Academy of Sciences, Beijing 100049

3 School of Geography & Ocean Science, Nanjing University, 163 Xianlin Street, Nanjing 210023, P.R. China.

4 Collaborative Innovation Center of Recovery and Reconstruction of Degraded Ecosystem in Wanjiang Basin Co-founded by Anhui Province and Ministry of Education, Anhui Normal University

*Correspondence: caiyj@niglas.ac.cn

Table S1 Macroinvertebrates dominant species in North the Lake Taihu at different stages

Stage	Species number	Dominant species
2007-2013	39	*Limnodrilus hoffmeisteri*
		Grandidierella taihuensis
		Corbicula fluminea
		Rhyacodrilus sinicus
		Tanypus chinensis
		Branchiura sowerbyi
		Potamilla acuminata
		Nephtys oligobranchia
		Notomastus latericeus
		Bellamya aeruginosa
		Dero sp.
		Chironomus flaviplumus
		Paranthura sp.
		Propsilocerus akamusi
		Boccardia proboscidea
		Microchironomus tabarui
		Sphaerium lacustre
		Semisulcospira cancelata
		Stenothyra glabra
		Proclakius choreus
2014-2019	37	*Limnodrilus hoffmeisteri*
		Corbicula fluminea
		Tanypus chinensis
		Grandidierella taihuensis
		Microchironomus tabarui
		Propsilocerus akamusi
		Paranthura sp.
		Branchiura sowerbyi
		Stenothyra glabra
		Bellamya aeruginosa
		Proclakius choreus
		Nephtys oligobranchia
		Cryptochironomus sp.
		Chironomus flaviplumus
		Sphaerium lacustre
		Nereis japonica
		Potamilla acuminata
		Semisulcospira cancelata

Glossiphonia complanata

Notomastus latericeus

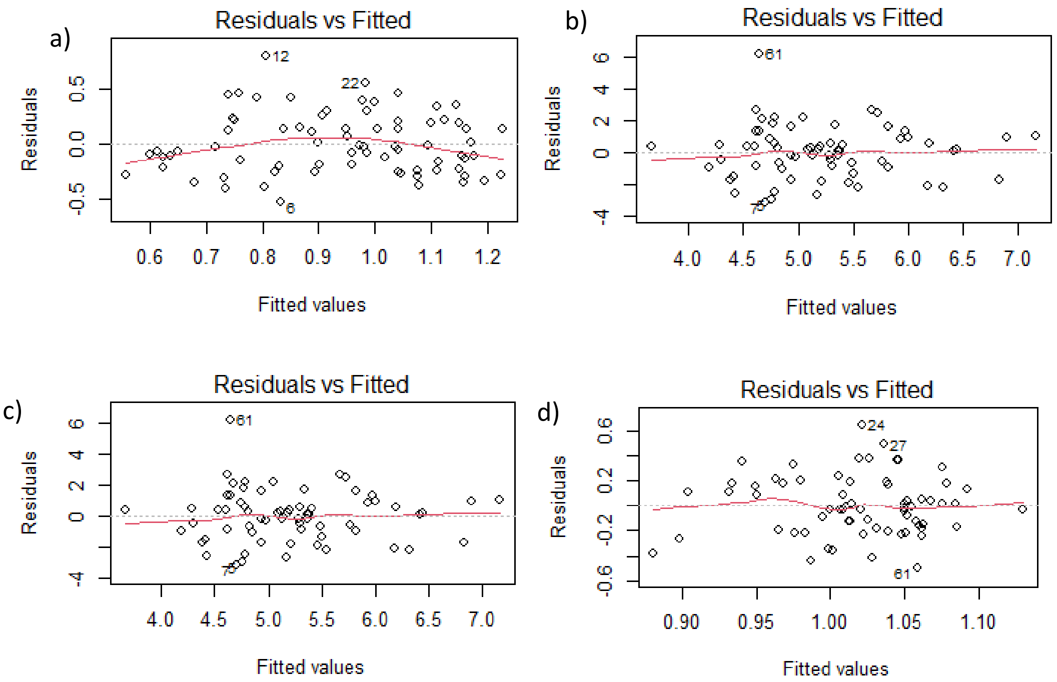

Figure S1 The Residuals vs Fitted plot between the density of all oligochaetes, the Shannon–Wiener index and the TLI index in northern Lake Taihu in different periods. (a: oligochaetes VS TLI index during 2007-2013; b: oligochaetes VS TLI index during 2014-2019; c: Shannon–Wiener index VS TLI index during 2007-2013; d: Shannon–Wiener index VS TLI index during 2013-2019)

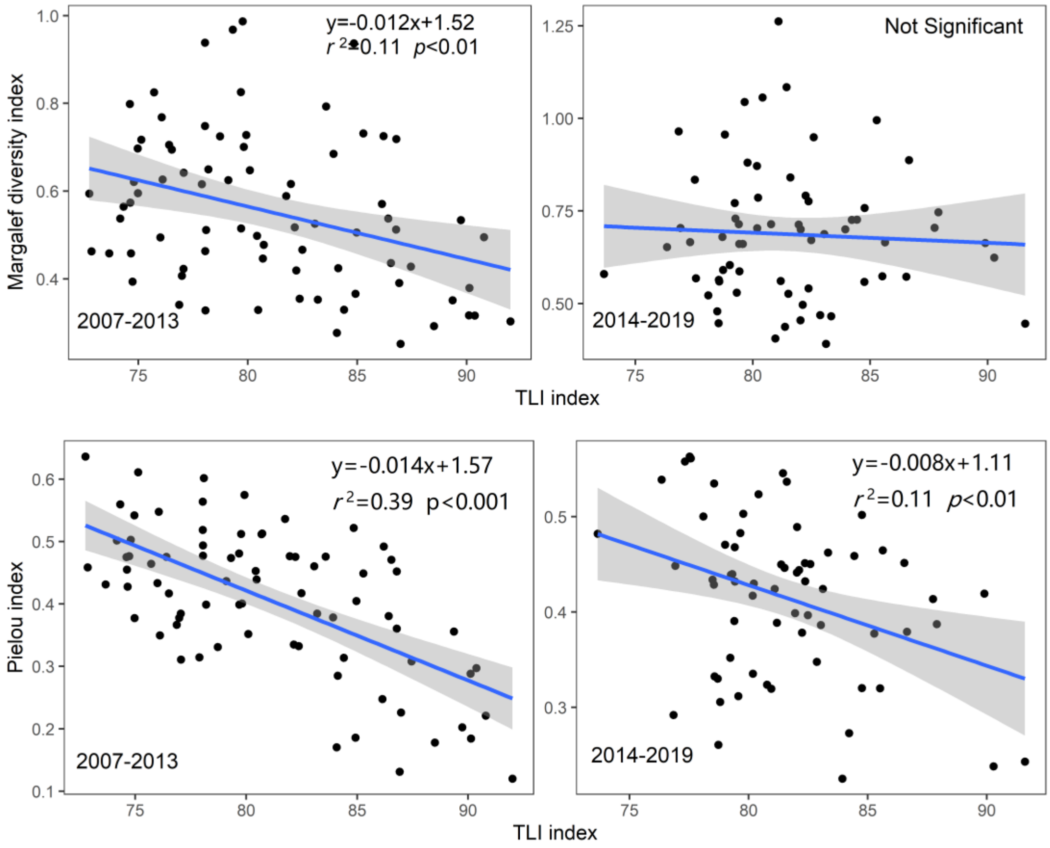

Figure S2 The relationship between the Pielou index and Margalef diversity index vs. the TLI index in North Taihu Lake in different periods.

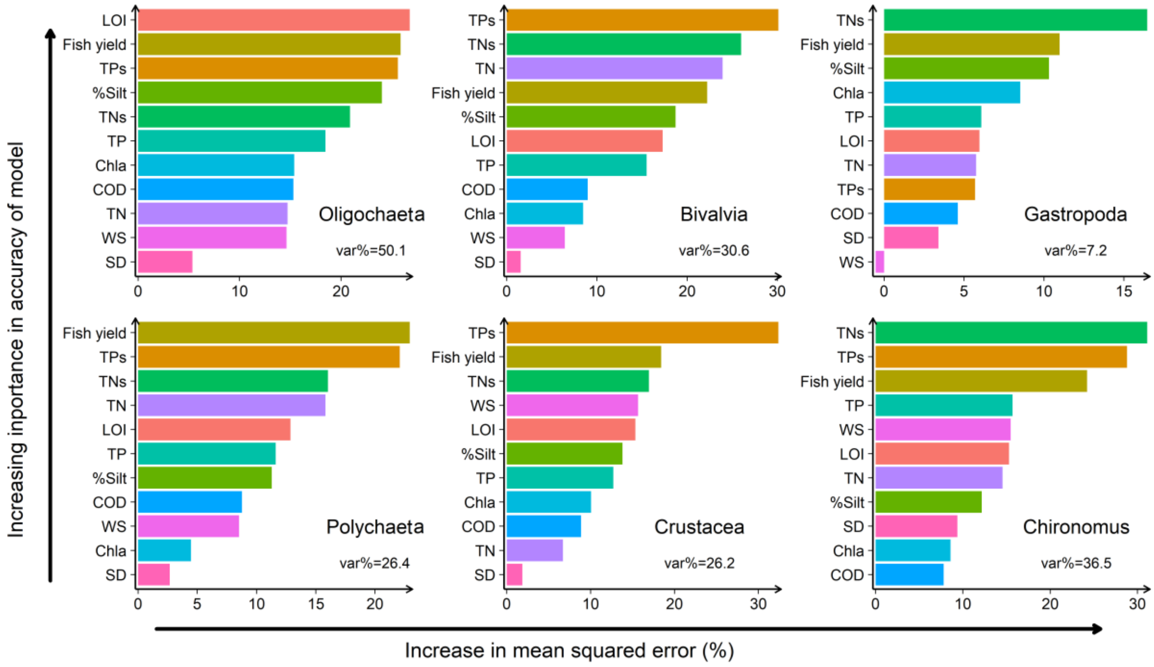

Figure S3 Ranking the importance of environmental factors on different classes of macroinvertebrates in the random forest model.
